# Healthcare and recreational sector collaboration strategies to support community-based physical activity participation among young people with childhood-onset physical disability: A scoping review protocol

**DOI:** 10.12688/hrbopenres.13961.1

**Published:** 2024-09-04

**Authors:** Karen Brady, Aoife Cooper, Ailish Malone, Nora Shields, Jennifer Ryan

**Affiliations:** 1 CP-Life Research Centre, School of Physiotherapy, RCSI University of Medicine and Health Sciences, Dublin, Ireland; 2 North Kerry Children’s Disability Network Team, Brothers of Charity Ireland, Listowel, Kerry, Ireland; 3 La Trobe University Olga Tennison Autism Research Centre, Melbourne, Victoria, Australia

**Keywords:** physical activity; childhood-onset disability; stakeholders; recreation; health

## Abstract

**Objective:**

The objective of this scoping review is to identify evidence of collaboration between healthcare and recreational sectors aimed at supporting community-based physical activity participation among young people with childhood-onset physical disability.

**Introduction:**

Most young people with physical disabilities do insufficient physical activity, significantly impacting their future health. There have been long outstanding calls for collaboration between healthcare and recreational professionals to support physical activity participation for people with disabilities. Given the importance of physical activity and the roles of health and recreational professionals, there is a need to systematically identify evidence on collaborative strategies between sectors, describe the experiences of all individuals involved in delivering and receiving these collaborations and describe any outcomes measured as part of implementing these strategies.

**Inclusion criteria:**

This review will include studies that involve healthcare professionals and recreational professionals working together to support community based physical activity. Specifically aimed young people aged 10 to 24 years with childhood-onset physical disabilities. Studies that report the experiences of individuals in delivering and receiving these collaborations will be included as well as studies that describe an evaluation of collaborative strategies.

**Methods:**

This scoping review will be conducted in accordance with the Joanna Briggs Institute methodology of scoping reviews. A comprehensive search strategy will be developed in consultation with an information specialist. The following databases will be searched: MEDLINE, CINAHL, Embase, Web of Science and Scopus. The review will consider studies of any design that address collaboration between health and recreation sectors including qualitative, quantitative and mixed-methods study designs. Two reviewers will independently screen each retrieved title and abstract and assess full-text articles against the inclusion criteria to determine eligibility. Data will be extracted and synthesized quantitively and qualitatively and mapped to a relevant framework.

## Introduction

Most young people with physical disability do insufficient physical activity ^1– 4^. This has significant implications for their future health ^5^. Despite the benefits and collective desire of healthcare professionals, families, recreational professionals, and young people themselves to enhance participation in physical activity, numerous barriers persist. Recreational professionals, such as personal trainers, gym staff, activity programme coordinators and sport coaches value the support of healthcare professionals to support community physical activity participation for people with disability and report a desire for collaboration ^6, 7^. Healthcare professionals can potentially bridge the gap between young people with disabilities, their families and community recreational professionals ^8^. However, they often face challenges in supporting and promoting physical activity due to limited knowledge and experience, accessible pathways, time constraints and conflicting priorities ^9^. Young people with disabilities and their families often struggle to find suitable activities, usually relying on word of mouth, while recreational professionals face challenges in reaching out to inform young people with disability about available physical activity programmes ^6^. These issues may be addressed through improved communication and collaboration between recreational and healthcare professionals. Since healthcare professionals frequently interact with young people and their families, enhancing their connection with recreational professionals may better support community-based physical activity. Young adults with cerebral palsy have prioritised cross sector collaboration strategies to optimise participation at community gyms with specific reference to the creation of pathways to the gym and involving healthcare professionals in training gym staff ^10^.

Recommendations for scientists and practitioners in both sectors to establish inter-professional communication channels and collaborate to address barriers have been reported ^11^. However, there is a need for efforts to develop, test and deliver collaborative strategies to improve physical activity participation, moving beyond describing the challenges and providing recommendations ^11^. Given the importance of physical activity and the roles of health and recreational professionals, synthesising existing evidence on collaborative strategies may support future research and practice to enhance community-based physical activity participation for young people with childhood-onset physical disabilities.

Therefore, the aim of this scoping review is to identify evidence of collaboration between healthcare and recreational sectors aimed at supporting community-based physical activity participation among young people with childhood-onset physical disability.

## Review objectives

1. Identify strategies that involve healthcare professionals and recreational professionals working together to support community-based physical activity participation among young people with childhood-onset physical disability
2. Describe the experiences of all individuals involved in delivering and receiving these collaborations, including healthcare professionals, recreational professionals, young people with disability and those who support them such as parents or personal assistants.
3. Describe the outcomes measured as part of implementing collaborative strategies
4. Consult with stakeholders to confirm the findings of the review, highlight unreported collaboration examples and experiences that have not been reported in the literature, and direct future collaborative strategies aimed at supporting community-based physical activity participation among young people with childhood-onset physical disability

## Inclusion criteria

### Participants

We will include studies with people aged 10–24 years, hereafter referred to as young people. This age-range encompasses ages included in definitions of adolescents and young people ^12, 13^. Where studies also include people outside of these age ranges, we will include them if the mean age is between 10 and 24 or if at least 50% of the sample is between 10 and 24. We will include people with any childhood-onset disability that primarily results in physical impairment, where the disability was either congenital or acquired before the age of 15 years. These may include cerebral palsy, spina bifida, spinal cord injury, acquired brain injury, traumatic brain injury, skeletal abnormality, brachial plexus injury, obstetric brachial plexus palsy, limb loss, neuromuscular disorder, multiple sclerosis, or genetic disorders in line with previous diagnostic categories reported ^14, 15^. We will include studies involving individuals with comorbidities such as intellectual disability and sensory impairment, provided they have a primary physical impairment. Where studies include a mixed population group, we will include them if at least 50% of the sample have a childhood-onset physical disability. When examining the experiences of collaborations, we will include studies with young people, healthcare professionals, recreational professionals, and people who support the young people including parents, other family members, disability support workers or personal assistants.

### Concept

Collaborative strategies implemented to support young people with childhood-onset physical disability to participate in community-based physical activity. This review will include studies that involve healthcare professionals and recreational professionals working together to support community based physical activity. If explicit evidence of collaboration is not available, we will accept the setting as indicative of collaboration, or look for explicit statements of partnerships, funding, or acknowledgments of cross-sector collaboration. The interaction could occur online or in person or over the phone at a single time-point or multiple time-points. Examples include but are not limited to healthcare professional contact with coaches or activity leaders, healthcare professionals instructing participants regarding exercises alongside recreational professionals, healthcare professionals educating or advising fitness professionals on disability, accessible pathways involving healthcare professionals and community organisers ^16^.

### Context

The aim of the collaborative strategy should be to support physical activity participation in the community. Community settings may include but not be limited to gyms, swimming pools, sports clubs, indoor and outdoor recreation centres. However, the interaction between healthcare professionals and recreational professionals does not have to occur in the community. Physical activity programmes or interventions that take place in a clinical setting will be included, if the aim of the study is to support community-based physical activity. Routine rehabilitation programmes, or programmes conducted in a participants home, education or employment setting will be excluded. We will include studies conducted in any country.

We will include studies published in any language. We will exclude studies published before 2000 because of the evolving perspectives on disability since 2000, such as the publication of the World Health Organization's International Classification of Functioning, Disability and Health (ICF) in 2001. This ensures our strategies stay relevant in light of current insights into disability and physical activity.

### Types of sources

The review will include studies of any design that explore collaboration between health and recreation sectors, including qualitative, quantitative, and mixed-methods studies. Quantitative studies may involve experimental (e.g., randomized and non-randomized trials) or observational (e.g., cohort, cross-sectional) designs. Excluded from the review are reviews, editorials, case reports, protocols, opinion papers, and conference abstracts

## Methods

This review will be conducted in accordance with the Joanna Briggs Institute methodology for scoping reviews and reported in line with the Preferred Reporting Items for Systematic Reviews and Meta-Analyses extension for Scoping Reviews (PRISMA-ScR) ^17, 18^.

### Search strategy

A comprehensive search strategy will be developed in consultation with an information specialist. First, an initial limited search of MEDLINE (PubMed) will be conducted to identify potentially relevant articles. Keywords from the title and abstract of relevant articles will inform the search strategy. Search terms will include keywords and index terms relating to childhood-onset disability, physical activity, healthcare and recreational professionals, and collaboration. The search strategy for MEDLINE (PubMed) is included (Appendix I). The search strategy will be modified as needed for the other databases. The following databases will be searched MEDLINE, CINHAL, EMBASE, Scopus and Web of Science. Reference list checking of included studies will take place 

### Study selection

Following the search, all identified publications will be collated and uploaded into Covidence with duplicates removed. A sample of randomly selected titles and abstracts will be independently screened by two reviewers, with results compared and discussed by the reviewers and further detail provided to eligibility criteria as needed. Two reviewers will then independently screen the titles and abstracts for inclusion. Full-text articles will be obtained and full-texts will be screened independently by two reviewers. Discrepancies will be resolved through discussion between the reviewers, and if consensus is not reached, a third reviewer will be consulted.

### Data extraction

Data will be extracted from the included studies independently by two reviewers using a modified version of the JBI data extraction template (Appendix II). Two reviewers will pilot the data extraction tool on at least two studies and make necessary amendments. Extracted data will include details about the population, concept, contexts, study design, methods, experiences, and outcomes relevant to the review questions. Authors of papers will be contacted for clarification or additional information if needed. In accordance with guidance on scoping review, a quality appraisal will not be conducted ^17^.

## Data analysis and presentation

Results of the literature search and study screening process will be presented in a PRISMA-ScR flow diagram ^18^. Narrative synthesis will describe collaborative strategies between healthcare and recreational professionals. We will report the identified strategies under the five pre-defined implementation strategies of the conceptual model of implementation research ^19^. The strategies will then be mapped to a relevant framework. The experiences of those delivering and receiving the collaboration will be analysed descriptively and based on the model's outcomes ^19^. The outcomes of implementing the collaborative strategies (participant outcomes or quantitative data) will be analysed descriptively. Our findings will be presented in tables or figures with three key headings: details of the collaborative strategy used, experiences with the strategy, and outcomes of its implementation.

## Knowledge user engagement

Engaging knowledge users is an important aspect of conducting scoping reviews. The latest JBI guidance recommends consultation at every stage of the scoping review, including topic prioritisation, planning, execution and dissemination, rather than limiting it to a single stage ^17^. In our proposed scoping review, knowledge users will contribute at each of these stages, highlighted below.

### Prioritisation and planning

Before conceptualising this scoping review, the research team established strong relationships with healthcare and recreational professionals who aim to better support community-based physical activity for young people with physical disabilities. Consultations at a World Café event with these professionals, young people with disabilities and their families highlighted the need for local and national collaboration between healthcare and recreational sectors “Children’s Disability Network Teams need to be connected and talking to sports organisations locally and nationally”.

### Execution and dissemination

Knowledge users from health and recreational sectors have approved the research question and reviewed the proposed protocol to ensure it aligns with the priority area identified through consultation. Their engagement will address review question four.

This may be achieved through a World Café event, an effective co-production method ^20^, or a similar opportunity where knowledge users can informally discuss the review's findings, highlight unreported collaboration examples and suggest dissemination strategies for the results. Knowledge users will be involved in the dissemination of the results and may include: development of evidence summaries or science communication strategies for social media, or advocate for findings to be shared in relevant community and political organisations to ensure they are reaching decision-makers.

## Ethics and consent

No Ethical approval or consent is required.

## Data Availability

No underlying data are associated with this article. Open Science Framework (OSF): Healthcare and recreational sector collaboration strategies to support community-based physical activity participation among young people with childhood-onset physical disability: A scoping review protocol https://doi.org/10.17605/OSF.IO/BPGZ3 This protocol has been registered prospectively on OSF and the registration includes a PDF document that provides the rationale for the review, the aim of the review, eligibility criteria, the search strategy, and plan for data extraction and data analysis and knowledge user engagement. A sample search strategy (Appendix I) and data extraction template (Appendix II) are included (subject to iterative changes). Extended data are available under the terms of the CC0 1.0 Universal licence
