# Supplementary material for: Healthcare and recreational sector collaboration strategies to support community-based physical activity participation among young people with childhood-onset physical disability: A scoping review protocol
Source: HRB Open Res. 2025 Mar 13;7:57. Originally published 2024 Sep 4. [Version 2] doi: 10.12688/hrbopenres.13961.2 (PMC12174905; doi:10.12688/hrbopenres.13961.2)
Supplement: Peer review history

# Reviewer report: Reviewer response for version 2

**DOI:** 10.21956/hrbopenres.15510.r47468

**Published:** 2025-06-17

**Authors:** Louise Declerck

**Affiliations:** 1 Kinesiology and Nutrition, University of Illinois Chicago College of Applied Health Sciences (Ringgold ID: 315410), Chicago, Illinois, USA; 2 UCLouvain, Louvain-la-Neuve, Walloon Region, Belgium

In light of the high levels of physical inactivity observed within the population of young people with childhood-onset physical disability, it is vital to identify implementation strategies that have successfully addressed this issue. Increasing collaborations between healthcare and recreational sectors is therefore an excellent avenue that requires further exploration. The research detailed within the protocol submitted by the authors aims aligns well with this. The evidence that will be synthesized in this scoping review will be highly relevant to the field. Some aspects of the protocol remain slightly unclear, and these have been summarized below:

-  
**Abstract:** The end of the abstract section states "Data will be extracted and synthesized quantitatively and qualitatively...". The use of the word "quantitatively" suggests that a meta-analysis and pooling of quantitative results will be performed. Unless I am mistaken, the complete methods does not mention this. Therefore, the abstract needs to be corrected to more accurately summarize how the data will be presented.

 

- **Inclusion criteria: **Consider justifying the thresholds you have used (10-24 years, disability acquired before 15 years old).Additionally, consider adding median as well as mean when stating "... we will include them if the mean age is between 10 and 24..."

 

- **Concept:** The sentence "If explicit evidence of collaboration is not available, we will accept the setting as indicative of collaboration..." is unclear and needs to be changed. In the examples included within this section, consider adding the situation whereby healthcare professionals directly refer a patient to a community-based exercise program, as this situation is, to my knowledge, one of the most common. 

 

- **Context:** "Physical activity programmes or interventions that take place in a clinical setting will be included, if the aim of the study is to support community-based physical activity." Though I understand this choice, I believe it may create some conflicts, as physical activity programmes provided in clinical settings are often (though not always) conducted by physical therapists and/or occupational therapists (so healthcare professionals), as opposed to coaches or activity leaders, as described previously in your method...Please describe what you will decide in such situations (-> include vs exclude).Please add the ICF's manual as reference when mentioning the ICF.

 

- **Data extraction**: How will discrepancies at the stage of quality assessment and data extraction be resolved?

 

- **Data analysis and presentation**: It would be very insightful to add more information regarding the five pre-defined implementation strategies of the conceptual model, for example in a table. This will allow a much better understanding of the data analysis plan. 

 

- **Knowledge user engagement:** If I understand this section correctly, you plan to collect additional data as well... Would that not require ethical approval from a board? And the adoption of a methodologically sound qualitative approach, for ex through focus groups or semi-structured interviews? Instead, I would perhaps say that: If the knowledge users highlight remaining research gaps in the literature, these will be subsequently investigated through future qualitative research.

 

- **Execution and dissemination:** Consider stating how many knowledge users from both the health and recreational sectors have validated your research question/aims/protocol.

I believe that modifying/adding this information would improve the overall quality of the protocol.

Is the study design appropriate for the research question?

Yes

Is the rationale for, and objectives of, the study clearly described?

Partly

Are sufficient details of the methods provided to allow replication by others?

Partly

Are the datasets clearly presented in a useable and accessible format?

Not applicable

Reviewer Expertise:

Post-doctorate research within the field of exercise training in neurorehabilitation.

I confirm that I have read this submission and believe that I have an appropriate level of expertise to confirm that it is of an acceptable scientific standard, however I have significant reservations, as outlined above.

# Reviewer report: Reviewer response for version 2

**DOI:** 10.21956/hrbopenres.15510.r47159

**Published:** 2025-06-12

**Authors:** Ming-Chih Sung

**Affiliations:** 1 California State University, Los Angeles, California, USA

This protocol outlines a planned scoping review aiming to map existing evidence of collaboration between healthcare and recreational sectors in supporting community-based physical activity participation among young people with childhood-onset physical disabilities. The review seeks to identify collaborative strategies, stakeholder experiences, and outcome measures related to these partnerships. 

 This is a scientifically sound and well-structured scoping review protocol addressing an important and timely topic in disability and physical activity research. The authors have made commendable revisions following peer feedback and have aligned the protocol with best-practice methodologies. However, addressing the minor but meaningful clarifications listed below will strengthen the protocol’s transparency and rigor.

 1. The term “healthcare professionals” is used repeatedly but remains undefined. The authors should clarify what constitutes a healthcare professional in this context—e.g., does this include only physicians and physiotherapists, or also occupational therapists, psychologists, nurses, etc.? This has implications for inclusion criteria and generalizability.

 2. The age range of 10–24 years is justified by aligning with common definitions of adolescence and youth. However, the exclusion of children aged 7–9 years, particularly those in primary school who may also benefit from collaborative physical activity support, seems arbitrary. The authors should either provide a rationale for this cutoff or reconsider including younger children or at least discuss this limitation explicitly.

 3. The authors state they will include studies in any language. While inclusive, they should briefly describe how they will ensure accurate translation and data extraction from non-English publications (e.g., will professional translation services or bilingual reviewers be used?).

Is the study design appropriate for the research question?

Yes

Is the rationale for, and objectives of, the study clearly described?

Yes

Are sufficient details of the methods provided to allow replication by others?

Partly

Are the datasets clearly presented in a useable and accessible format?

Not applicable

Reviewer Expertise:

I am a faculty member in the School of Kinesiology, with research focused on promoting physical activity and motor behavior among individuals with disabilities. My work also includes evidence synthesis, such as systematic reviews, scoping reviews, and meta-analyses.

I confirm that I have read this submission and believe that I have an appropriate level of expertise to confirm that it is of an acceptable scientific standard, however I have significant reservations, as outlined above.

# Reviewer report: Reviewer response for version 2

**DOI:** 10.21956/hrbopenres.15510.r46375

**Published:** 2025-03-20

**Authors:** Kwok Ng

**Affiliations:** 1 Physical Activity for Health Research Centre, Department of Physical Education and Sport Sciences, University of Limerick, Limerick, County Limerick, Ireland

The authors have thoroughly responded to the earlier queries. I believe they have updated the protocol adequately to help them with their intended search and acceptance.

Is the study design appropriate for the research question?

Yes

Is the rationale for, and objectives of, the study clearly described?

Partly

Are sufficient details of the methods provided to allow replication by others?

Yes

Are the datasets clearly presented in a useable and accessible format?

Not applicable

Reviewer Expertise:

I'm an academic researcher in adapted physical activity and evidence synthesis Ireland fellow.

I confirm that I have read this submission and believe that I have an appropriate level of expertise to confirm that it is of an acceptable scientific standard.

# Reviewer report: Reviewer response for version 1

**DOI:** 10.21956/hrbopenres.15317.r42799

**Published:** 2024-11-11

**Authors:** Kwok Ng

**Affiliations:** 1 Physical Activity for Health Research Centre, Department of Physical Education and Sport Sciences, University of Limerick, Limerick, County Limerick, Ireland

The area of healthcare professions' role in promoting PA among adolescents with disabilities is an important one. The authors have chosen a good topic to carry out a scoping review, as it would broaden the knowledge than just effectiveness that would come from a meta analyses. The authors could more clearly the reason and justification for doing a scoping review. It is not clear. 

 Also, I would like to have seen authors used established definitions of 'people with disabilities' and then go into the concept of physical disability, how healthcare workers promoting PA differs from people from other impairment groups, and outline clearly which disability model is being used. This may be a useful exercise for the authors to consider the keywords, inclusion/exclusion criteria, and world cafe, and reporting from the review. 

 Here are some comments on the research objectives that the authors may like to consider responding to. 

 The research objective is to 'identify strategies', it would be good to know what lens is being used to identify a 'strategy' over a 'programme', 'technique', 'actions', 'policies', etc. Authors should outline a clear definition of what makes a strategy, and use frameworks that have helped to define strategies over other aspects.

 2nd research objective is a bit vague. I believe a more narrow focus is more feasible in a scoping review. how do you plan to 'describe the experiences'. This was not mentioned clearly in the methods or even in the introduction to suggest there is a need for this. The diverse group of individuals that the experiences would be described from is very broad, and authors could outline how they plan to disseminate this without diluting the information.

 3rd research objective seems incomplete. Kindly outline it, and take into note of more detail required from 2nd objective.

 4th objective, as I understand it, they are doing this as part of the scoping review, through the world cafe. Overall, that would fit without an objective to carry out a scoping review, and following JBI recommendations. To me, that would not be needed to be outlined as an objective. Rather, if there is more that would be done, , then it can be included. 

Is the study design appropriate for the research question?

Yes

Is the rationale for, and objectives of, the study clearly described?

Partly

Are sufficient details of the methods provided to allow replication by others?

Yes

Are the datasets clearly presented in a useable and accessible format?

Not applicable

Reviewer Expertise:

I'm an academic researcher in adapted physical activity and evidence synthesis Ireland fellow.

I confirm that I have read this submission and believe that I have an appropriate level of expertise to confirm that it is of an acceptable scientific standard, however I have significant reservations, as outlined above.

# Response

**Published:** 2025-03-07

**Authors:** Karen Brady

**Affiliations:** Royal College of Surgeons in Ireland, Dublin, Leinster, Ireland

We thank the reviewer for the constructive feedback on this protocol. We now address each point in responses below the review comment

 The area of healthcare professions' role in promoting PA among adolescents with disabilities is an important one. The authors have chosen a good topic to carry out a scoping review, as it would broaden the knowledge than just effectiveness that would come from a meta-analyses. The authors could more clearly the reason and justification for doing a scoping review. It is not clear.

**Response:**

 According to Joanna Briggs Institute (JBI) methodology  guidelines, scoping reviews help identify the types of available evidence, existing knowledge gaps, clarify key concepts or definitions, assess research trends  and inform future systematic reviews. 

 Our research question aims to identify evidence of collaboration between healthcare and recreational providers supporting community-based physical activity for child-onset physical disability. A scoping review was chosen as the most appropriate method for addressing this question. This will allow the existing evidence to be mapped, key knowledge gaps identified, and the key characteristics of collaboration to be described , following JBI guidelines.

 Also, I would like to have seen authors used established definitions of 'people with disabilities' and then go into the concept of physical disability, how healthcare workers promoting PA differs from people from other impairment groups, and outline clearly which disability model is being used. This may be a useful exercise for the authors to consider the keywords, inclusion/exclusion criteria, and world cafe, and reporting from the review.

**Response:**

 The United Nations Convention on the Rights of Persons with Disabilities defines individuals with disabilities as “those who have long-term physical, mental, intellectual, or sensory impairments which, in interaction with various barriers, may hinder their full and effective participation in society on an equal basis with others.” This definition provides a broad understanding of disability, but we recognise the challenge of balancing inclusivity with the need for specificity, given the individuality of disability.

 Disability encompasses a wide range of conditions, each presenting unique challenges. For instance, physical disabilities primarily affect a person’s mobility or physical functioning. However, individuals with physical disabilities may have more functional similarities than differences. Law et al. (2004) found that young people with physical disabilities exhibit similar participation levels, regardless of their specific diagnosis. This suggests that an approach  based on function, rather than one based solely on diagnosis, may be effective. For this study, we have chosen to focus on young people with child-onset physical disabilities, which may include conditions such as cerebral palsy, spina bifida, or neuromuscular disorders, provided the impairments first appeared before the age of 15. This definition aligns with the approach used by McKenzie et al. (2021) in their reporting on child-onset physical disabilities.

 Here are some comments on the research objectives that the authors may like to consider responding to.

 The research objective is to 'identify strategies', it would be good to know what lens is being used to identify a 'strategy' over a 'programme', 'technique', 'actions', 'policies', etc. Authors should outline a clear definition of what makes a strategy, and use frameworks that have helped to define strategies over other aspects.

**Response:**

 We recognise the need for greater clarity regarding the definition of strategies and have further expanded on this now. In Proctor’s 2013 conceptual model of implementation research, strategies represent the "how-to" aspect, with Curran (2012) defining implementation strategies as “methods or techniques used to enhance the adoption, implementation, and sustainability of a clinical programme or practice.” 

 2nd research objective is a bit vague. I believe a more narrow focus is more feasible in a scoping review. how do you plan to 'describe the experiences' . This was not mentioned clearly in the methods or even in the introduction to suggest there is a need for this. The diverse group of individuals that the experiences would be described from is very broad, and authors could outline how they plan to disseminate this without diluting the information.

**Response:**

 We consider  our 2nd objective to be appropriate for a scoping review where the purpose is to help identify the types of available evidence, existing knowledge gaps, clarify key concepts or definitions, assess research trends  and inform future systematic reviews.  Other reviews such as systematic reviews are more appropriate to address precise narrow effectiveness questions.

 We plan to describe the experiences through qualitative research. Qualitative research is a type of research that explores and provides deeper insights into real-world problems ( Moser et al 2017). A qualitative approach allows participants themselves to explain how, why, or what they were thinking, feeling, and experiencing at a particular time or during an event of interest. It answers the hows and whys instead of how many or how much (Tenny et al 2022). Therefore, we believe that it is important to report the experiences of the professionals collaborating because understanding their experiences can provide valuable insights into what worked, what didn’t, and what may need to change or adapt to improve the implementation and sustainability of these partnerships. Data describing the experiences of professionals will be extracted from qualitative studies. 

 Equally important is capturing, the experiences of young people and their families regarding these collaborative efforts. Depending on the available evidence, our synthesis may include descriptions of if / how the collaboration between sectors benefited them; if it made participation easier; and if it addressed barriers to participation.  Gathering this feedback can help identify ways to enhance support for young people and their families moving forward. Data describing the experiences of young people and their families will be extracted from qualitative studies.

 3rd research objective seems incomplete. Kindly outline it, and take into note of more detail required from 2nd objective.

**Response:**

 Objective 3 has been modified to “Identify and describe any outcomes measures that were used to evaluate the collaboration strategy used by healthcare and recreational providers”

 As outlined above, we have now provided further clarity for ‘strategy’ as per Curran 2012 “methods or techniques used to enhance the adoption, implementation, and sustainability of a clinical programme or practice.” 

 4th objective, as I understand it, they are doing this as part of the scoping review, through the world cafe. Overall, that would fit without an objective to carry out a scoping review, and following JBI recommendations. To me, that would not be needed to be outlined as an objective. Rather, if there is more that would be done, , then it can be included.

**Response:**

 Upon reflection, we agree that this does not need to be an objective. Following JBI recommendations for scoping reviews, stakeholder engagement is important. We will incorporate this consultation throughout the review process, rather than making it a separate objective, and it has been integrated into the knowledge user engagement section of the protocol.
